# Turnover and Turnover Intention Among Nurses Working in Saudi Arabia: A Qualitative Evidence Synthesis

**DOI:** 10.1111/jan.16875

**Published:** 2025-03-14

**Authors:** Sarh Almubark, Andrew Booth, Emily Wood

**Affiliations:** ^1^ Population Health, School of Medicine and Population Health The University of Sheffield Sheffield UK; ^2^ Health Services Management Department, Faculty of Public Health Umm Al‐Qura University Makkah Kingdom of Saudi Arabia

**Keywords:** leadership, literature review, non‐Saudi nurses, nurses, nursing, personnel turnover, retention, review, Saudi Arabia, turnover intentions

## Abstract

**Aims:**

This review's primary objective is to explore factors causing turnover and turnover intention in nurses working in KSA and to identify ways to prevent turnover and reduce turnover intention in the KSA nursing workforce.

**Design:**

Qualitative evidence synthesis (QES).

**Data Sources:**

MEDLINE/Ovid/PubMed, Web of Science, PsychINFO, CINAHL, and Google Scholar (GS) underwent a structured search for articles. Articles were selected for inclusion if they reported primary studies with qualitative or mixed methods study designs published in English or Arabic in the peer‐reviewed literature or as a thesis or dissertation.

**Review Methods:**

In order to determine which type of synthesis to choose, we applied the RETREAT framework recommended in the Cochrane handbook and used by other researchers. Thematic synthesis was the most applicable choice, so this approach was selected.

**Results:**

Seven studies published in nine reports in the years 2016 through 2022 were included. The final coding framework included five predominant themes related to 19 subthemes. Three main findings were that there are leadership challenges at all levels in the KSA healthcare system leading to nurse turnover, a complex web of discrimination discourages nurses from remaining in the Saudi healthcare workforce, and societal pressure experienced by both Saudi and non‐Saudi nurses leads to turnover and turnover intention.

**Conclusions:**

KSA leaders should focus on intervening in the leadership challenges found at all levels of the KSA healthcare system. Addressing this issue could also positively impact the related issues of discrimination and societal pressure in the workplace and could begin to take steps toward improving occupational conditions and reducing nurse turnover and turnover intention.

**Impact:**

Addressing the serious problem of the leadership challenges in healthcare would likely have a strong positive impact on the other two findings that relate to discrimination and societal pressure.

**Patient or Public Contribution:**

Not applicable.

**Summary**

- What is already known
  ○ Most of the nurses serving in the health system in the Kingdom of Saudi Arabia (KSA) are non‐Saudis.
  ○ KSA faces challenges with the recruitment and retention of both Saudi and non‐Saudi nurses, who have different needs.
  ○ Evidence‐based recommendations for improved recruitment and retention of both Saudi and non‐Saudi nurses are lacking.
- What this paper adds
  ○ After reviewing nine reports of seven qualitative studies on the topic, five predominant themes were identified.
  ○ First, it is recommended that KSA address an overall lack of leadership in the healthcare system.
  ○ Second, it is recommended that KSA healthcare leaders directly address the complex web of workplace discrimination.
  ○ Finally, KSA leaders can work to ease the impact of societal pressures on non‐Saudi workers.

## 1 Introduction

Keeping clinical facilities staffed with nurses is a worldwide problem. In a 2021 meta‐analysis, researchers identified a total of 18 cross‐sectional surveys representing 23 countries of intensive care unit (ICU) nurse turnover intention and found that the pooled prevalence rate of turnover intention was 27.7% (Xu et al. 2023). Nurse shortages impact different countries differently, with nurses migrating away from less favourable working conditions in one country to countries with more resources offering more opportunities (Drennan and Ross 2019).

Nurse turnover in hospitals in the Kingdom of Saudi Arabia (KSA) has been studied for about the last 10 years because it is an ongoing problem that accelerated during the COVID‐19 pandemic (Alsadaan et al. 2021; Alzahrani 2022; Falatah 2021). The results from several quantitative studies show that when job satisfaction is low, turnover intention increases (Albougami et al. 2020; Al‐Dossary et al. 2012; Alshareef et al. 2020; Falatah and Conway 2019). Also, negative experiences in the work environment, such as low quality‐of‐life (QoL) at work, bullying at work, or having a supervisor with poor leadership skills have also been shown to measurably increase turnover intention in many studies (Al Muharraq et al. 2022; Al‐Ahmadi 2014; Albougami et al. 2020; Almalki et al. 2012a, 2012b; Al‐Mansour 2021; Alshareef et al. 2020; Soqair 2021; Suliman et al. 2020).

Like every country, the Kingdom of Saudi Arabia (KSA) needs nurses. The KSA healthcare system is comprised of a large public infrastructure alongside a growing private one (Albougami et al. 2020; Almalki et al. 2011). The public sector is primarily managed by the Ministry of Health (MoH) which includes primary, secondary, and tertiary healthcare facilities providing services free of charge to Saudi citizens. On the other hand, the private sector provides healthcare services for both Saudi and non‐Saudi patients on a fee‐for‐service basis (Al‐Hanawi et al. 2020). Although KSA has its unique challenges, it essentially is competing in the global nursing shortage for nurses (Falatah and Salem 2018).

An important consideration when examining the nursing workforce in KSA is pathways to employment for Saudis vs. non‐Saudis. According to Alluhidan et al. (2020), as of 2018, there was a total of 184,565 nurses in KSA, but only 70,319 (around 38%) were Saudi citizens. Approximately 62% of the nurses working in KSA are female, but 90% of non‐Saudi nurses are female, reflecting the cultural value in KSA for wanting a same‐sex nurse for care (Alluhidan et al. 2020). Non‐Saudi nurses are considered guest workers, and their employment is arranged through agencies; these individuals are predominantly Indian, Filipino, and Malaysian (Alluhidan et al. 2020). Therefore, they have different needs than Saudi nurses, including requiring salary, housing, transportation, and other support as defined in an employment contract (Alluhidan et al. 2020).

Nurses who are Saudis are less likely to be staff nurses and more likely to be in leadership, even though there are few Saudi nurses trained as advanced practice nurses (APRNs) (Alluhidan et al. 2020; Alreshidi et al. 2021). Also, Saudi nurses are more likely to work for MOH health facilities compared with non‐Saudi nurses (Alluhidan et al. 2020).

The last 10 years of research into this issue have revealed differences between the needs of Saudi vs. non‐Saudi nurses working in KSA, and therefore, factors leading to turnover and turnover intention are slightly different in these two populations. One survey of non‐Saudi nurses at eight MOH hospitals recruited by WhatsApp (*n* = 639) had 97% female respondents, and 72% were staff nurses (meaning very few leaders) (Alreshidi et al. 2021). In that study, over 82% had a bachelor's degree, and 67% had at least 5 years of experience (Alreshidi et al. 2021). The largest ethnic groups were 44% Filipino and 50% Indian (Alreshidi et al. 2021). However, most studies over the last 10 years do not acknowledge that these two groups have completely different needs from a nursing work environment and profession, and so they fail to stratify their analyses by these two groups (Al‐Mansour 2021; Alshareef et al. 2020; Soqair 2021).

Many studies have been conducted of different nurse populations in KSA, and many of the same factors have been reported as leading to turnover intention, such as poor working conditions, low pay, and lack of opportunity for advancement (Falatah and Salem 2018). Further, nurses working in KSA may have personal needs and demands, such as for child care or for job security, that are not being met by the employment arrangement (Al‐Ahmadi 2014; Aljohani and Alomari 2018; Soqair 2021). A recent survey of a 1200 bed tertiary hospital in Riyadh found that almost 80% of the nurses were non‐Saudi, which is consistent with estimates from other recent studies (Al Muharraq et al. 2022).

Even with these studies, certain gaps remain in the literature. It is not clear what factors disproportionately influence Saudi compared to non‐Saudi nurses in terms of turnover and turnover intention. This is because these groups are often analysed together, so separate findings are not reported. Although many articles exist about nurse turnover in nurses working in many different countries, including KSA, there is no summary of what has been found specifically about KSA with respect to turnover and turnover intention, and how factors leading to turnover may differentially impact Saudis compared to non‐Saudis.

## 2 Aims

The primary objective of this qualitative evidence synthesis is to explore factors causing turnover and turnover intention in nurses working in KSA in both Saudi and non‐Saudi nurses. It is to identify ways to prevent turnover and reduce turnover intention in the KSA nursing workforce among both Saudi and non‐Saudi nurses by identifying and synthesising themes from the results of qualitative studies on this topic to provide a deeper understanding of the unique challenges faced by both Saudi and non‐Saudi nurses.

## 3 Methods

### 3.1 Selection of Qualitative Evidence Synthesis (QES) Design

The QES is one approach to reviewing studies that is systematic, but it has the limitation of only being able to include qualitative studies (Flemming et al. 2019). However, this focus on qualitative data makes it particularly suitable for this study, as it allows for the synthesis of data to provide rich insights that align with the research questions.

### 3.2 Search Methods for Identification of Studies

The intention of the search strategy was to be comprehensive, and the single country focus made this feasible. MEDLINE/Ovid/PubMed, Web of Science, PsychINFO, CINAHL, and Google Scholar (GS) underwent a structured search for articles. The final electronic search strategies were developed using these key terms: (“Nurses” OR “Nursing staff” OR “Nursing workforce” OR “Registered nurses” OR “Saudi Arabia nurses”) AND (“Nurse turnover” OR “turnover” OR “Nurse retention” OR “retention” OR “Job satisfaction” OR “Nurse turnover intention” OR “Turnover retention”) AND (“Workplace experience” OR “Job experiences” OR “Organisational culture” OR “Work‐life balance” OR “Career satisfaction” OR “Job stress” OR “Prevent turnover” OR “Reduce turnover intention”) AND (“Qualitative research” OR “Grounded theory” OR “Phenomenological research” OR “Ethnographic study” OR “Case study” OR “Qualitative analysis”). In MEDLINE/Ovid/PubMed, Web of Science, PsychINFO, and CINAHL, these were executed as keyword searches, because that type of search looks through all fields of the citation record, including title and abstract (Falagas et al. 2008). By contrast, GS is programmed to be a keyword search, so in GS, only keyword searching was used (Falagas et al. 2008; Gehanno et al. 2013).

Databases generally are programmed to do a “smart search” where keywords like “nurse” are searched along with other iterations of the same word, such as “nursing” and “nurses” (Falagas et al. 2008). GS is considered the gold standard for searching and definitely employs this approach in their searches (Falagas et al. 2008; Gehanno et al. 2013). Because the search was executed in all these databases, including GS, it is believed that no sources were missed. The following date range was searched with these limits applied: Years 2016 through 2022.

Search criteria were entered into these databases, and all the pages of the results were reviewed manually by one author (SA) to evaluate inclusion and exclusion criteria. This author retrieved the full text of all items identified as potentially relevant and placed them in an electronic library. A different author (AB) re‐executed the search strategy and validated the screening and selection process. The co‐authors assessed these items independently and resolved disagreements by discussion and did not need to involve a third party. We agreed on study selection.

First, abstracts and titles were reviewed, and articles that clearly did not meet inclusion and exclusion criteria were ruled out. Next, the remaining articles underwent full‐text screening for inclusion and exclusion criteria, and a final determination was made.

Where the same study, using the same sample and methods, had been presented in a peer‐reviewed article and a thesis or dissertation, we chose the thesis or dissertation version of the study and excluded the article version of the study. This was to ensure study representation was unbiased and that the unit chosen provided the most primary data.

### 3.3 Selection Criteria

The search strategy was informed by the SPIDER (Sample, Phenomenon of Interest, Design, Evaluation, Research type) tool, rendering the following search terms: “Saudi Arabia” nurse, turnover, retention, job satisfaction, qualitative (Cooke et al. 2012).

Table 1 documents the SPIDER method used.

**TABLE 1:** SPIDER results.

Item	Search terms
Sample	nurse, working in Saudi Arabia
Phenomenon of interest	nurse turnover, reasons for nurse turnover, nurse turnover intention, nurse job satisfaction, nurse retention
Design	thematic, ethnography, framework synthesis, phenomenology
Evaluation	attitudes, perception, opinion, suggestion, recommendation, experience
Research type	qualitative, mixed‐method

From the results of the SPIDER tool (Cooke et al. 2012) in Table 1, the selection criteria were developed. The dates chosen for searching were from 2016 to 2022, when the research was conducted. The date 2016 was chosen because it marks the beginning of Vision 2030, a countrywide strategic plan in KSA (Alsufyani et al. 2020).

The topic of interest was turnover and turnover intention in nurses working in KSA. As part of this, the topics of job satisfaction, working environment, nurse retention, and related topics were studied as they relate to turnover and turnover intention in nurses working in KSA. Due to the implementation of Saudi Vision 2030 in 2016, the items included in the review were published between the year 2016 and the last complete year (2022) (Kingdom of Saudi Arabia 2017). Items included reported primary qualitative studies or mixed‐methods studies with a qualitative component. The participants in the studies were nurses working in KSA at any level (managers, staff, etc.). This included native Saudis as well as non‐Saudi nurses, as long as they were working in any healthcare setting in Saudi Arabia when the study was done. We did not exclude studies based on our assessment of methodological limitations. We instead used this information about methodological limitations to assess our confidence in the review findings.

We included primary studies that use qualitative study designs, or a qualitative study design as part of a larger mixed‐methods study. The qualitative study designs could include ethnography, phenomenology, case studies, grounded theory studies, and qualitative process evaluations. We included studies that use both qualitative methods for data collection (e.g., focus group discussions, in depth interview, observation, diaries, document analysis, open‐ended survey questions) and qualitative methods for data analysis (e.g., thematic analysis, framework analysis, grounded theory). We excluded studies that collect data using qualitative methods but do not analyse these data using qualitative analysis methods (e.g., open‐ended survey questions where the response data are analysed using descriptive statistics only).

We included published studies in the peer‐reviewed literature or theses, or dissertations. They can be published in either English or Arabic. We included mixed methods studies where it is possible to extract the data that were collected and analysed using qualitative methods.

### 3.4 Data Collection and Analysis

Qualitative evidence synthesis aims for variation in concepts rather than an exhaustive sample, and large amounts of study data can impair the quality of the analysis (Booth et al. 2018). Once we identified all studies that were eligible for inclusion, we assessed whether their number or data richness was likely to represent a problem for the analysis, in which case we would consider selecting a sample of studies. However, the country‐specific scope suggested at the design stage that a comprehensive sample for Saudi Arabia may be feasible to review in this qualitative evidence synthesis.

**FIGURE 1:**
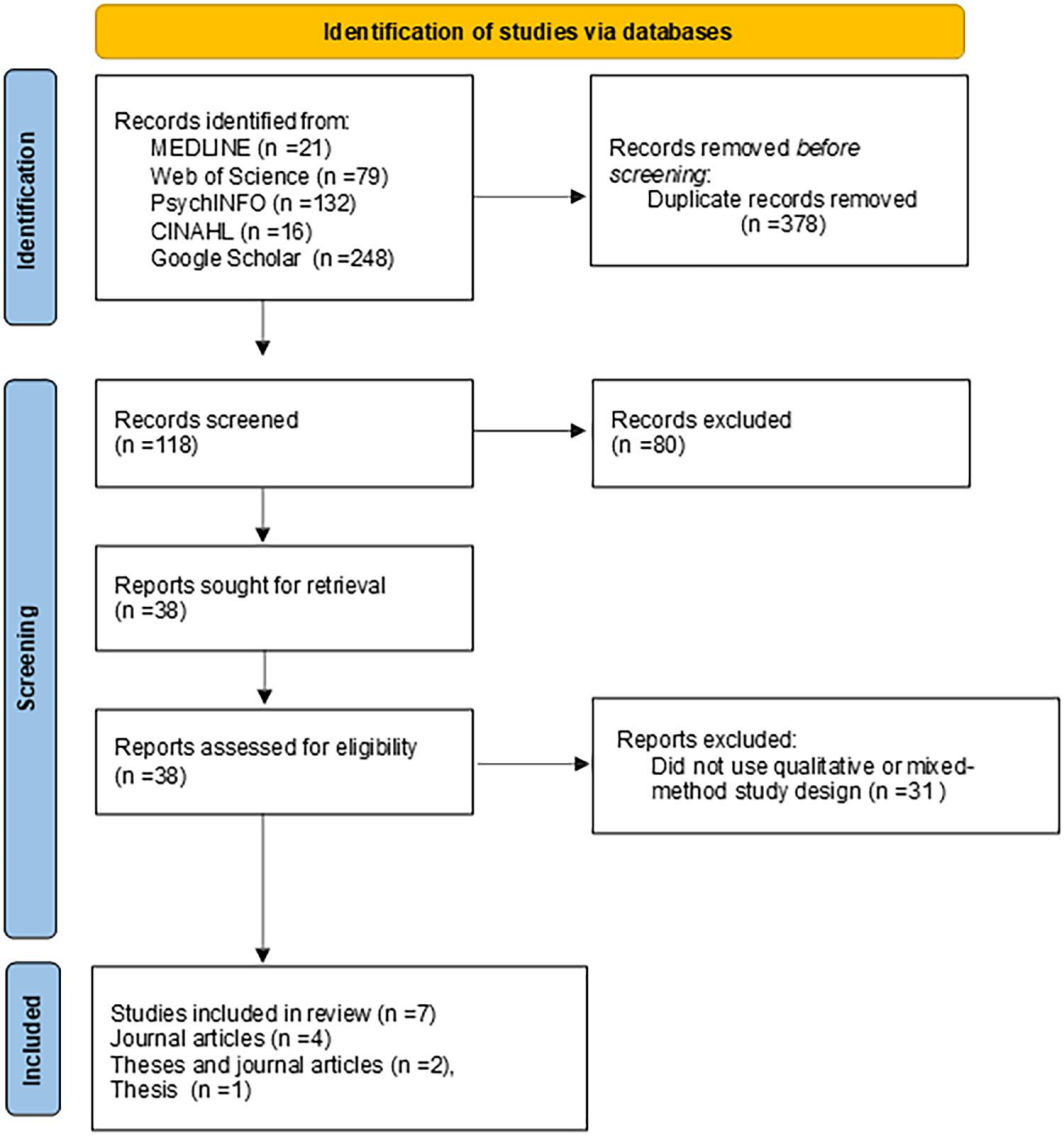
Includes the PRISMA flow chart.

As shown in Figure 1, seven articles were selected.

First, data was collected about each study. These data points included: Title, authors, year of publication, what database they were present in, type of publication (thesis/dissertation which were identified in GS or peer‐reviewed journal article), study design (qualitative or mixed‐methods with a qualitative component), method of data collection, and whether or not a framework was used for study design. Post hoc, the following data were collected: Setting, number of participants, participant characteristics, and data collection method.

Next, original verbatim text extracts from the studies of inclusion were copied from the studies and placed on an Excel spreadsheet. If original verbatim text extracts were not available and only themes were available, these were extracted and placed on the spreadsheet.

The method chosen to assess the methodological limitations of included studies was adapted from several articles. First, the original article on the Critical Appraisal Skills Programme (CASP) was reviewed (Long et al. 2020), along with evidence from the literature related to Cochrane reviews and qualitative research (Flemming et al. 2019; Noyes et al. 2018). Next, how the CASP was adapted for use in a particular study was reviewed (Karimi‐Shahanjarini et al. 2019). To address the unique characteristics of qualitative research, it was decided to adapt the modified CASP tool to assess methodological limitations (Malpass 2009). The reviewer was asked to classify the article based on seven items that are supposed to be completely addressed in each article in order for it to have a high methodological appraisal. After all seven items were classified on all articles, an overall assessment of the methodological limitations was done (see Appendix A).

Two authors (SA and AB) independently reviewed each article and completed the CASP tool. Next, these authors met for a consensus conference, and the reviews were accepted.

### 3.5 Data Management, Analysis and Synthesis

In order to determine which type of synthesis to choose—framework, thematic, or meta‐ethnography—we applied the RETREAT framework recommended in the Cochrane handbook and used by other researchers (Booth et al. 2018). A thematic synthesis seemed to be the most applicable choice, so this approach was selected. We synthesised themes using the approach for thematic qualitative data analysis described by Burnard et al. (2008). The data collection was done by the author (SA). Briefly, first, direct quotations from respondents reported under the heading “results” of each included study were transferred to an Excel spreadsheet. These verbatim text extracts were transferred along with information about the themes on which they were placed in the original studies. Next, for each study, an initial coding framework was developed. Specifically, a code was developed for each theme expressed in each verbatim text extract, and these were coded for each study. The data was recorded in an Excel spreadsheet.

Data were collected from journal articles first, and from theses second. This was to make data collection efficient, in that each successive data collection sought to standardise the themes being identified. Saturation of themes took place during the analysis. Once this analytic step was complete, there was an initial coding framework for all studies of inclusion. At that stage, the identified themes were merged into a final coding framework, which will be presented in the results section as the final synthesis. Not all the included studies used a framework.

### 3.6 Assessing Our Confidence in the Review Findings

Two review authors (SA and AB) used the GRADE‐CERQual (Confidence in the Evidence from Reviews of Qualitative research) approach to assess our confidence in each finding (Lewin et al. 2018). GRADE‐CERQual assesses confidence in the evidence based on the following four key components.

1. Methodological limitations of included studies: the extent to which there are concerns about the design or conduct of the primary studies that contributed evidence to an individual review finding.
2. Coherence of the review finding: an assessment of how clear and cogent the fit is between the data from the primary studies and a review finding that synthesises those data. By cogent, we mean well supported or compelling.
3. Adequacy of the data contributing to a review finding: an overall determination of the degree of richness and quantity of data supporting a review finding.
4. Relevance of the included studies to the review question: the extent to which the body of evidence from the primary studies supporting a review finding is applicable to the context (perspective or population, phenomenon of interest, setting) specified in the review question.

After assessing each of the four components, we made an assessment of the overall confidence in the evidence supporting the review finding. We judged the confidence as high, moderate, low, or very low. The final assessment was based on consensus among the review authors (see Appendix B). All findings started as high confidence and were then graded down if there were important concerns regarding any of the GRADE‐CERQual components.

## 4 Results

After criteria were applied, nine articles representing seven studies were included in the analysis, arranged according to the year of publication. The articles were all in English. Table 2 summarises studies of inclusion, while additional details can be found in the Table S1.

**TABLE 2:** Studies of inclusion.

ID	First author	Year	Study design	Setting	Number of participants	Participant characteristics	Qualitative data collection method	Data analysis method
1	Alotaibi	Article: (2016)	Quantitative non‐experimental, descriptive research	Seven hospital sites, each of which represented a different region of Saudi Arabia	271	Saudi nurses at hospital sites (e.g., non‐Saudi nurses excluded)	Open‐ended questions on a questionnaire	Thematic
2	Almansour	Thesis: (2017) Article: (2022)	Mixed methods	Three major government hospitals in Saudi Arabia	26	Nurses selected from different nationalities, including Saudi	Semi‐structured interview	Thematic
3	Aljohani	Article: (2018)	Mixed methods	Saudi Ministry of Health recruitment office	124	Filipino nurses applying to work in Saudi	Short answer on a questionnaire	Thematic
4	Saleh	Article: (2018)	Qualitative	Medical city in Saudi Arabia	35	Saudi and non‐Saudi nurses working at the institution	Semi‐structured interview	Thematic
5	Alshareef	Thesis: (2019) Article: (2020)	Mixed methods	Hospitals in Jeddah and Mecca cities	245	All nurses employed at these sites were sent an anonymous survey	Open‐ended questions on a questionnaire	Thematic
6	Shatnawi	Thesis: (2020)	Mixed methods	Two hospitals in Riyadh	19	All Intensive care nurses (Saudi and non‐Saudi)	Semi‐structured interview	Thematic
7	Al‐Nusair	Article: (2022)	Mixed methods	Major private medical/surgical hospital in Saudi Arabia	20	Immigrant nurses	Semi‐structured interview	Thematic

*Note:* Further information on the studies included is available in the Table S1.

As shown in Table 2, four of the studies were published as journal articles only (1, 3, 4 and 7), two were published as both theses and journal articles (2 and 5), and the remaining study was published only as a thesis (6).

Table 3 presents the themes and subthemes from the Final Coding Framework.

**TABLE 3:** Themes and subthemes from the final coding framework.

Theme	Subthemes
Relationships with supervisors/leads	Leadership interactions Management support
Workplace equity	Perception of nursing profession Concepts of seniority Involvement in decision‐making Salary Professional disrespect Opportunities for educational/career development Personnel policies Social support
Work assignments/workload	Night and weekend shifts Emotions caused by work Language of communication at work Job satisfaction
Quality of life	Living accommodations Recreational activities Work transportation
Saudi society	Islamic/Saudi norms and policy Family support

As shown in Table 3, there were five main themes, and each one comprises between two and nine subthemes. The process of deriving the themes was inductive. These themes and subthemes are explained below. However, as a preface to this section, it is important to distinguish between the terms “leadership” and “management” Leadership could be seen as having vision, inspiration, and passion, and using these traits to lead teams, while management could be seen as the act of creating and managing processes that are aimed at maximising efficiency and delegating authority (Aleksoski et al. 2020). Therefore, leaders fail at leadership if they fail to have vision, inspiration, and passion, and managers fail at management if they do not create and manage efficient processes and do not delegate authority well.

### 4.1 Relationships With Supervisors/Leads

The first theme captures relationships between supervisors or leads and their reports, and includes two subthemes: leadership interactions and management support. In all of the included studies, the relationships between supervisors/leads and subordinates were consistently reported to be poor. Staff described constant leadership conflicts and felt a lack of support from management:

There is a gap between us and our head nurse; she does not listen to us. (Alotaibi et al. 2016).

It's stressful situation because…there is lack of support from the management that the staff can't be protected by the organization with the lack of support. (Shatnawi 2020).

As acknowledged previously, “leadership” and “management” are different concepts. A healthcare setting could have excellent leaders who are poor managers, and vice versa. It may be that management is easier than leadership, and therefore, there are fewer challenges. This review found however ample evidence of both extensive management and leadership complications in the Saudi healthcare environment.

### 4.2 Leadership Interactions

Although these results cover seven different studies, it is important to point out that one of them, by Saleh et al. (2018), focuses almost exclusively on the dominance of ineffectual leadership styles seen in the Saudi healthcare system and how they impact nursing. This study focused on how these ineffectual leadership styles appear to be common and complicate the caregiving environment in which nurses operate (Saleh et al. 2018).

I wish the head nurse can be proactive…just don't listen to one particular group of people, but to everyone…I know it's difficult. (Saleh et al. 2018).

Nevertheless, as shown under the Management Challenges section, the other studies reviewed additionally provide ample evidence of serious complications with leadership throughout the Saudi healthcare system.

One common issue with leadership, expressed independently of management issues, is that professional medical colleagues are not expected to treat nurses with respect.

Another weak point here in this country, is that the doctors just give us orders and instruction and never look at us as colleagues, and [the doctors and other leadership] look at us as lower than them and assume they are the superior and the leader… (Shatnawi 2020).

If we have an issue, we are not allowed to go talk to the directors…There is a chain of command. They're getting worse and worse; there are no changes… (Almansour 2017).

As evidenced by these verbatim extracts, in general, leadership would allow and even cultivate this environment of professional disrespect. Unfortunately, this professional disrespect also in some instances would metastasize into racism.

Unfair leadership dominant by race. (Alshareef 2019).

### 4.3 Management Support

As mentioned earlier, one study focused exclusively on leadership style, and that is because, although management and leadership are different concepts, they are inextricably linked (Saleh et al. 2018). If a manager has difficulty executing the most basic management tasks, it is unlikely they will be able to be a successful leader. Such examples of management failures were abundant in the studies reviewed. First, there were instances of managers abusing nurses.

My boss controls us, he does not manage us. I am being manipulated and psychologically abused by my boss. Every meeting is a chance for him to treat us badly, depending on his mood… I hate coming to work. (Almansour 2017).

My nurse manager embarrasses us, and if someone makes even a little mistake, everyone will be disciplined, which is unfair…I left that area because of her. (Almansour 2017).

Next, there were instances of managers not protecting nurses who were abused.

I feel very bad and stressed because sometimes you can't accept that kind of abuse you face from family with the lack of support from the hospital… (Shatnawi 2020).

…my colleagues working with me at night duty has been abused and that time I called the supervisor to explained it to patient by Arabic but what he has done? Nothing… (Shatnawi 2020).

Another theme in the area of poor management was not showing the nurses that they were appreciated.

Well I never received an appreciation letter, nor have I ever been appreciated…the doctors get appreciated even though the nurse plays a key role and that is unfair…that really upsets me and makes me depressed and think about leaving this hospital. (Shatnawi 2020).

I never received any thank you letter or been recognised even I am a hard worker… (Shatnawi 2020).

Other cases of poor management that were less severe centred around setting up an inefficient workplace, and ineffectively delegating.

…we are not well staffed and sometimes you feel that you need to have some extra staff with the extra tasks we are having… But the management refuse and we manage it ourselves. (Shatnawi 2020).

My nurse manager is unfair…My head nurse prefers her friends in assigning weekends off. She is unsupportive and discriminatory. (Almansour 2017).

Although these verbatim text extracts present evidence of management failures in the Saudi healthcare workplace, in many places, the evidence also demonstrates leadership failures. Managers who treat nurses abusively, or do not protect them from occupational risks, are not even fulfilling the basic management objectives. Those who are able to fulfil basic management objectives may lack skills, and be inefficient at routine tasks such as scheduling and ensuring proper patient flow. This lack of management in this setting leads to high levels of stress as reported by these nurses.

#### 4.3.1 Workplace Equity

Workplace Equity was a main concern in all of the included studies, as nurses at all levels reported multiple types of discrimination. This theme comprised eight subthemes.

### 4.4 Perception of Nursing Profession

The first subtheme, which relates to how the nursing profession is perceived, captures how some in Saudi society look down on nurses and do not think Saudi women should be involved in that profession.

The image of female Saudi nurses is still low; most people still do not have enough respect for them (Alotaibi et al. 2016).

### 4.5 Concepts of Seniority

The second subtheme relates to the concept of seniority in the workplace, or that experience in a field should lead to greater credit and credentials. Staff nurses in included studies complained of having a lot of experience in the Saudi nursing context, but not receiving any credit for their experience:

There is no value placed on sincerity and skills (Alshareef 2019).

### 4.6 Involvement in Decision‐Making

The third subtheme under the Workplace Equity theme describes involvement in decision‐making. Nurses at all levels who were interviewed for included studies complained that they were often shut out of decision‐making, either by physicians or administrative leaders.

The main factors that influenced his decision to leave his job was not sharing in decision‐making (Alshareef 2019).

### 4.7 Salary

A fourth subtheme is linked to salary; in the studies, nurses of all levels uniformly reported challenges with low salary. Importantly, they describe how nurses could achieve a higher salary by playing racial politics. One nurse described a preference for nurses from Canada and the US, saying that they are paid a higher salary for the same work.

… there are different categories of salaries depending on from where you come. If you come from Canada and the US, your salary is top‐top… I think that is not fair. (Almansour 2017).

Two nurses expressed intention to turnover simply because of this salary discrimination:

The salary is not fair compensation between we Filipinos and other nationalities. I am going to Europe or Canada. (Almansour 2017).

Many expressed the need for leadership to step in and fix the problem of salaries being based up on race or ethnicity, and that this was a prominent reason to consider leaving the Saudi healthcare system as a workplace:

Salary scale and benefits should be same for all nationalities and should only be fixed on the basis of position but not nationality… Salary enhancement should be standardised… [nor] on the basis on ethnicity, race and nationality. (Alshareef 2019).

### 4.8 Professional Disrespect

Another subtheme conveys how nurses relate to professional disrespect in the workplace context. Participants in the studies reviewed consistently reported poor relationships with physicians:

Another weak point here in this country, is that the doctors just give us orders and instruction and never look at us as colleagues, and they look at us as lower than them and assume they are the superior and the leader. That's the worst thing and makes you stressful because such a relationship can affect the care provided to patients. And this stress is added to your job and your day. (Shatnawi 2020).

Sometimes this professional disrespect was directed to nurses from nurses. One nurse expressed her disgust at the unprofessional favouritism displayed by her manager:

My nurse manager is unfair… My head nurse prefers her friends in assigning weekends off. She is unsupportive and discriminatory. (Almansour 2017).

Finally, certain sets of policies and norms comprise the final three subthemes of the Workplace Equity theme.

### 4.9 Opportunities for Educational/Career Development

First, nurses emphasised the subtheme of opportunities for work‐related education and career development throughout the studies and consistently complained that these were lacking in the Saudi context.

I have a Diploma of Nursing and there is not one university within Saudi Arabia that will consider my previous study… (Alotaibi et al. 2016).

### 4.10 Personnel Policies

Next, nurses complained that personnel policies related to pregnancy and childbirth make it difficult to start a family, making them consider suspending their work in the profession during childbearing.

There is not any consideration for working mothers during the pregnancy period, [or any] maternity leave or leave for sick days for kids. Moreover, there is no day care for the babies to make it easy for the mothers to work (Alshareef 2019).

### 4.11 Social Support

Finally, as many of these nurses are ex‐patriots, policies that lead to social support are very important to them. Throughout the included studies, nurses complained consistently about the lack of social support in the workplace:

If you are alone and you feel pressure at work, you do not have anyone to express it to…I wanted to go as I missed my family in Malaysia… (Almansour 2017).

#### 4.11.1 Work Assignments/Workload

This theme relates to how work assignments and workload are distributed among the nurses in the Saudi healthcare workplace. Throughout the studies, nurses repeatedly reported unequal or excessive work assignments and workloads. This theme relates to four subthemes.

### 4.12 Night and Weekend Shifts

The first subtheme relates to night and weekend shifts, which nurses prefer to avoid because the work is more stressful during those shifts. In the included studies, nurses repeatedly reported unfairness in the distribution of these shifts among the staff, in that Saudis complain about being assigned to work these shifts:

…the night shift is hard for me and my family. (Almansour 2017).

Most Saudi nurses want to work morning duties. They do not want to work at night. This is a problem… (Almansour 2017).

### 4.13 Emotions Caused by Work

The second subtheme is emotions caused by work, which in this case were all in the negative direction toward work‐related stress and burnout. Work‐related stress was mentioned frequently in the verbatim text extracts in the studies reviewed.

I have no time to take my break completely, the unit is always full and the patients here are very sick and busy, this caused me high level of stress all the times. (Shatnawi 2020).

### 4.14 Language of Communication at Work

Another subtheme related to this theme relates to the language of communication used at work. The official language in the workplace is English, and though many individuals from all over the world convene in the Saudi healthcare workplace, they are all expected to communicate in English. In the studies reviewed, non‐Saudi nurses expressed frustration with the lack of fluency of Saudi nurses in English and complained that it was hard to communicate with them in English at work.

…We have our job descriptions, but teaching English is not part of our job description. We have become teachers, and it creates more work for us (Almansour 2017).

### 4.15 Job Satisfaction

Finally, job satisfaction was mentioned over and over in the studies reviewed. When the topic came up, the tone was always negative, in that nurses were experiencing a lack of satisfaction in their jobs:

There are not enough nurses so we have a lot of work to do, which decreases job satisfaction (Alotaibi et al. 2016).

#### 4.15.1 Quality of Life

The fourth primary theme was quality of life, and whenever it was discussed, it was in the negative. Three areas where nurses repeatedly reported lack of or low quality of life in the included studies contribute the three subthemes to this theme: living accommodation, recreational activities, work transportation, and other facilities or provisions.

### 4.16 Living Accommodations

Non‐Saudi nurses must rely on their employer for living accommodation, and those nurses complained that these were consistently less than adequate.

First, it's really the accommodations…We have problems. The washing machine did not work for months, and we washed our clothes manually. Finally, I bought my own [washing machine] as if you waited for the machine to be repaired, it would take months (Almansour 2017).

The problem is the accommodations. The room should be for only two [occupants], but we [have] three… (Almansour 2017).

### 4.17 Work Transportation

Non‐Saudi nurses also complained about transportation issues, such as delays that disrupt their schedules.

The biggest problem for us is transportation. They [the coaches] are always late. They do not come on time… (Almansour 2017).

### 4.18 Recreational Activities

In terms of recreational activities, Saudi society has traditionally not cultivated the types of public recreational activities seen in Western countries, so non‐Saudis may find themselves bored. Although it is changing currently, even Saudi nurses who have studied abroad agree that there can be low quality of life in Saudi due to a lack of recreational activities. One even stated that having more recreational activities available would probably prevent turnover:

We don't have recreation activities. It is not like at other hospitals… They have a lawn tennis court…which I can truly say would help encourage people to stay (Almansour 2017).

No extracurricular activities, no gym and no swimming pool (Alshareef 2019).

#### 4.18.1 Saudi Society

Saudi society is unique in many ways, but is generally considered conservative and traditional, with strict cultural norms. Because these cultural norms are so strict, even modern Saudis often feel pressure as a result of them, which is the final theme identified.

### 4.19 Islamic/Saudi Norms and Policy

Saudi nurses complained of societal pressure for them to live up to certain ideals while performing as a nurse, while non‐Saudi nurses complained of societal pressure from a country whose norms were not completely consistent with theirs.

… I do feel that I have been discriminated against as a female in the country as I can't go out any time and I can't drive either, and that is what really stresses me out (Shatnawi 2020).

### 4.20 Family Support

For the non‐Saudis, an important subtheme was family support, in that they felt especially isolated from their family if the family could not join them in Saudi. The pressure from cultural Saudi norms made this separation more difficult. Societal norms and practices arising from Islam were identified as a second subtheme and a source of pressure on these nurses. While many were Muslim themselves, those who were not felt uncomfortable participating in Muslim practices such as wearing an abaya.

We women feel inferior, we cannot drive, and we need to wear an Abaya (covering all the body and hair with a special dress and head cover) and cannot go out alone. We need to be accompanied by our relative and at the same time; we are not allowed to bring our family because of visa restrictions and because of feeling of homesick and missing family members. (Shatnawi 2020).

## 5 Discussion

This qualitative evidence synthesis revealed three findings: leadership challenges, complex webs of discrimination, and Saudi societal factors place pressure on Saudis and non‐Saudis (see Appendix B). These overall findings were expressed as five themes and 19 subthemes relating to factors causing turnover and turnover intention in nurses working in KSA. Most of the participants interviewed in the qualitative analyses included in this review reported many challenges in the healthcare occupational setting in KSA that would encourage nurse burnout and turnover and discourage recruitment and retention.

This review, however, looks beyond nursing to the systemic issues in the KSA healthcare workplace reported first‐hand by the nurses in these studies. While it was known that poor nursing management was one important cause of nurse turnover in KSA (Soqair 2021), this review illustrates how management in KSA healthcare appears to have broken down at all levels, not just in nursing. Also, while discrimination and bullying have been documented in nursing in KSA (Al Muharraq et al. 2022; Alsadaan et al. 2021), this review illustrates how complex and multi‐faceted these systems of discrimination and bullying really are, and how difficult they may be to dismantle. Finally, while it was known that non‐Saudi nurses experience societal pressure when in KSA that can increase turnover intention (Alsadaan et al. 2021), this review illustrates a more complex impact of the culturally restrictive Saudi society on all nurses. While non‐Saudi nurses complain of having to conform to Saudi society, even Saudi nurses reported a conflict between workplace demands and societal expectations.

Our conceptual framework provided valuable insights in exploring nurse turnover, capturing nuances that traditional summaries often overlook. This framework enabled us to integrate diverse findings systematically, revealing patterns and connections not previously recognised. Through this framework, we identified significant factors that contribute to nurse turnover, enriching our overall understanding. To that end, after reviewing the themes and the findings, any interventions aimed specifically at reducing nurse turnover in KSA in the last decade have essentially failed, and this is likely because systemic change is needed to correct this problem (Albougami et al. 2020; Alonazi and Omar 2013; Soqair 2021). This systematic change could start at the bottom—with each individual healthcare leader at each organisation. If all current leaders in healthcare were encouraged to undergo retraining to gain the necessary skills needed for dismantling these systemic problems, this would be the first step toward reform (De Brún et al. 2019).

Although much evidence exists in the literature that exposes and explains the drivers of nurse turnover and turnover intention, few evidence‐based studies of interventions to reduce nurse turnover and turnover intention are available for consideration. A recent realist review recommended the following solutions to be implemented by nurse leaders: place emphasis on fostering social connectedness, provide the environment for professional practice autonomy, use methods to cultivate a healthy workplace culture, and identify ways to support professional growth and development (Cardiff et al. 2023). The articles included in this review were all observational in that they did not test any interventions (Cardiff et al. 2023). A study from South Korea assembled a focus group of nurses to provide suggestions for ways to reduce turnover (Yun and Yu 2021), and other writing in the literature consists of thought leaders proposing potential solutions or doing demonstration projects (Clemmons‐Brown 2023; Perkins 2021). Alshahrani (2022) recommended general solutions for nurse turnover in KSA, including changing the negative image of nursing, improving nurse working conditions, improving wages, and finding ways to retain aging nurses. However, no results from evidence‐based, practical interventions were included (Alshahrani 2022). A recently published doctoral dissertation reports the findings of a qualitative study where US nurses were asked about successful turnover reduction strategies (Cozart 2024). The participants recommended improving employee communication, motivating lower‐level nurses by making them a priority, improving organisational culture, and having competitive wages (Cozart 2024).

### 5.1 Policy Implications

The findings suggest that KSA healthcare leaders should rethink how management is trained and how healthcare is organised, given the leadership challenges identified in this review. Addressing the serious problem of the management challenges in healthcare would likely have a strong, positive impact on the other two findings that relate to discrimination and societal pressure. Having top‐tier managers working with healthcare clinicians and staff would improve the working conditions immensely and may simply reduce discrimination through strong leadership at all levels. Also, high‐quality managers have strategies to reduce stress in the workplace and could help both Saudi and non‐Saudi workers feel more comfortable in the face of societal pressures.

Knowing this, we would recommend that KSA implement a Leadership Development Program at the countrywide level. These programs should provide training in areas such as effective management skills and strategic thinking. Additionally, these programs should support continued education to prepare nurse leaders with the resources needed for addressing workplace challenges and promoting a positive workplace environment. Implementing these programs would empower nurses and encourage their professional growth and career advancement. Furthermore, KSA should promote recreational activities for non‐Saudi nurses within the healthcare organisation to enhance their well‐being. Such a nationwide program for nurses would be consistent with KSA's current strategic plan called Vision 2030, which seeks to reform the healthcare system to meet the growing demands of an aging and expanding population (Alluhidan et al. 2020).

## 6 Limitations

This qualitative evidence synthesis is not without limitations. First, although the quality of research methods was comparable across all the studies included, ultimately, study quality was not perfect. As shown in Appendix A, out of seven articles, three had major methodological flaws, and this likely impacted this paper's findings. These studies—three of which were in thesis format, so were dissertation topics—lacked a succinct summary of what was found. This led to challenges in synthesising all the themes from the different items. Also, the qualitative evidence was different from studies that were mixed methods and included surveys with open‐ended questions compared to studies where the data was obtained from interviews.

## 7 Conclusions

In conclusion, this qualitative evidence synthesis revealed three overarching findings related to the causes of turnover and turnover intention in nurses working in KSA. This setting includes a mix of Saudis and non‐Saudis who follow different pathways to employment. Further, the background Saudi culture places specific types of societal pressure on both Saudi and non‐Saudi nurses.

These findings call for KSA leaders to address overall leadership challenges at all levels in healthcare, to intervene on serious problems with discrimination in the workplace, and to take action to reduce the societal pressure felt by both Saudi and non‐Saudi nurses in the occupational setting. Any interventions over the past decade to reduce turnover and turnover intention in nurses working in KSA likely failed due to the persistent poor conditions in the overall KSA healthcare working environment. While KSA leaders are strongly encouraged to intervene on these systemic issues, it is recommended that they prioritise improving leadership and professionalism at all levels of the healthcare system first. By focusing on alleviating the leadership challenges, interventions by KSA leaders may also result in a positive impact on discrimination and societal pressure in the workplace.

## Conflicts of Interest

The authors declare no conflicts of interest.

## Supporting information


Table S1.

## Data Availability

The data supporting the findings of this study were obtained from publicly available databases, including, MEDLINE/Ovid/PubMed, Web of Science, PsychINFO, CINAHL, and Google Scholar.

## Appendix A The Assessments in the Methodological Limitations

**Table:**

Methodological limitations	
✓	None
Minor	Minor
?	Moderate
×	Major

Study ID	Year	Was the context described?	Was the sampling strategy appropriate and described?	Was the data collection strategy appropriate and described?	Was the data analysis appropriate and described?	Were the findings supported by evidence?	Is there evidence of researcher reflexivity?	Have ethical issues been taken into consideration?	Overall assessment of methodological limitations
Alotaibi	2016	?	×	✓	?	✓	×	✓	Moderate
Almansour	2017	✓	?	✓	✓	✓	×	✓	Minor
Aljohani	2018	?	×	×	?	×	×	?	Major
Saleh	2018	✓	×	✓	✓	?	×	?	Moderate
Alshareef	2019	✓	×	×	×	✓	×	✓	Major
Shatnawi	2020	✓	✓	✓	✓	✓	✓	✓	None
Al‐Nusair	2022	?	✓	✓	?	×	×	?	Major

## Appendix B GRADE‐CERQual Findings

**Table:**

Summary of review finding	Studies contributing to the review finding	Methodological limitations	Coherence	Adequacy	Relevance	GRADE‐CERQual assessment of confidence in the evidence	Explanation of GRADE‐CERQual assessment
**Leadership challenges:** Due to the economic and policy environment, workers at all levels in the healthcare system in Saudi lack leadership and management skills. This includes staff nurses, head nurses, nurse managers, physicians, higher level managers, and members of the administration, and is true of both Saudis and non‐Saudis	1–7	Moderate methodological limitation (1 study with minor, 2 studies with moderate, and 3 studies with major methodological limitations)	No or minor concerns about coherence	No or minor concerns about adequacy	Minor concerns	High confidence	Data from 9 items reporting 3 studies with major limitations and minor concerns about coherence and adequacy
**Complex webs of discrimination:** Unequal treatment of workers happens at all levels for complex reasons relating to favouritism, nepotism, stereotypes, racial and ethnic discrimination (especially Saudi vs. non‐Saudi), sexism, favouritism toward certain professions, and other features depending upon the local context and makeup of the employees in it	1–7	Moderate methodological limitation (1 study with minor, 2 studies with moderate, and 3 studies with major methodological limitations)	No or minor concerns about coherence	No or minor concerns about adequacy	Minor concerns about relevance (Most items reported at least one type of discrimination, but did not assign overall discrimination as a theme or finding)	High confidence	Data from 9 items reporting 3 studies with major limitations and minor concerns about coherence and adequacy
**Saudi societal factors place pressure on Saudis and Non‐Saudis:** Saudis feel societal pressure to adhere to Saudi and Islamic norms, but the workplace environment makes this difficult. Non‐Saudis feel oppressed by Saudi norms, such as having to wear and abaya, and having lack of recreation in Saudi	2 and 5–7	Major methodological limitation (1 studies with moderate, and 2 studies with major methodological limitations)	No or minor concerns about coherence	No or minor concerns about adequacy	Minor concerns about relevance (This finding is connected with the first two findings, and will have a strong influence over any solutions proposed. Therefore, this finding is highly relevant)	High confidence	Data from 1study with moderate limitation 2 studies with major limitation and minor concerns about coherence and adequacy

## References

[jan16875-bib-0001] Al Muharraq, E. H. , O. G. Baker , and S. M. Alallah . 2022. “The Prevalence and the Relationship of Workplace Bullying and Nurses Turnover Intentions: A Cross Sectional Study.” SAGE Open Nursing 8: 23779608221074655. 10.1177/23779608221074655. 35097205 PMC8796075

[jan16875-bib-0002] Al‐Ahmadi, H. 2014. “Anticipated nurses' Turnover in Public Hospitals in Saudi Arabia.” International Journal of Human Resource Management 25, no. 3: 412–433. 10.1080/09585192.2013.792856.

[jan16875-bib-0003] Albougami, A. S. , J. U. Almazan , J. P. Cruz , et al. 2020. “Factors Affecting Nurses' Intention to Leave Their Current Jobs in Saudi Arabia.” International Journal of Health Sciences 14, no. 3: 33–40. PMC7269627 32536847

[jan16875-bib-0004] Al‐Dossary, R. , J. Vail , and F. Macfarlane . 2012. “Job Satisfaction of Nurses in a Saudi Arabian University Teaching Hospital: A Cross‐Sectional Study.” International Nursing Review 59, no. 3: 424–430. 10.1111/j.1466-7657.2012.00978.x. 22897196

[jan16875-bib-0005] Aleksoski, O. , A. Stojanovska‐Stefanova , and M. Magdinceva Sopova . 2020. “Management Versus Leadership in the Modern World.” SocioBrains, International Scientific Refereed Online Journal With Impact Factor 7: 74.

[jan16875-bib-0006] Al‐Hanawi, M. K. , S. Almubark , A. M. N. Qattan , A. Cenkier , and E. A. Kosycarz . 2020. “Barriers to the Implementation of Public‐Private Partnerships in the Healthcare Sector in the Kingdom of Saudi Arabia.” PLoS One 15, no. 6: e0233802. 10.1371/journal.pone.0233802. 32555648 PMC7302438

[jan16875-bib-0007] Aljohani, K. A. , and O. Alomari . 2018. “Turnover Among Filipino Nurses in Ministry of Health Hospitals in Saudi Arabia: Causes and Recommendations for Improvement.” Annals of Saudi Medicine 38, no. 2: 140–142. 10.5144/0256-4947.2018.140. 29620549 PMC6074360

[jan16875-bib-0008] Alluhidan, M. , N. Tashkandi , F. Alblowi , et al. 2020. “Challenges and Policy Opportunities in Nursing in Saudi Arabia.” Human Resources for Health 18: 98. 10.1186/s12960-020-00535-2. 33276794 PMC7716289

[jan16875-bib-0009] Almalki, M. J. , G. Fitzgerald , and M. Clark . 2011. “Health Care System in Saudi Arabia: An Overview.” Eastern Mediterranean Health Journal 17, no. 10: 784–793. 22256414 10.26719/2011.17.10.784

[jan16875-bib-0010] Almalki, M. J. , G. FitzGerald , and M. Clark . 2012a. “Quality of Work Life Among Primary Health Care Nurses in the Jazan Region, Saudi Arabia: A Cross‐Sectional Study.” Human Resources for Health 10, no. 1: 30. 10.1186/1478-4491-10-30. 22971150 PMC3543175

[jan16875-bib-0011] Almalki, M. J. , G. FitzGerald , and M. Clark . 2012b. “The Relationship Between Quality of Work Life and Turnover Intention of Primary Health Care Nurses in Saudi Arabia.” BMC Health Services Research 12, no. 1: 314. 10.1186/1472-6963-12-314. 22970764 PMC3507760

[jan16875-bib-0012] Almansour, H. 2017. “The Association Between Nationality, Job Satisfaction and ‘Intention to Leave’ Among Nurses in Saudi Arabian Government Hospitals.” Phd thesis, University of Southampton. https://eprints.soton.ac.uk/429748/.

[jan16875-bib-0013] Al‐Mansour, K. 2021. “Stress and Turnover Intention Among Healthcare Workers in Saudi Arabia During the Time of COVID‐19: Can Social Support Play a Role?” PLoS One 16, no. 10: e0258101. 10.1371/journal.pone.0258101. 34618851 PMC8496805

[jan16875-bib-0015] Alonazi, N. A. , and M. A. Omar . 2013. “Factors Affecting the Retention of Nurses: A Survival Analysis.” Saudi Medical Journal 34, no. 3: 288–294. 23475094

[jan16875-bib-0016] Alotaibi, J. , P. S. Paliadelis , and F.‐R. Valenzuela . 2016. “Factors That Affect the Job Satisfaction of Saudi Arabian Nurses.” Journal of Nursing Management 24, no. 3: 275–282. 10.1111/jonm.12327. 26260125

[jan16875-bib-0017] Alreshidi, N. M. , L. M. Alrashidi , A. N. Alanazi , and E. H. Alshammeri . 2021. “Turnover Among Foreign Nurses in Saudi Arabia.” Journal of Public Health Research 10, no. 1: 1971. 10.4081/jphr.2021.1971. 33849251 PMC8054764

[jan16875-bib-0018] Alsadaan, N. , L. K. Jones , A. Kimpton , and C. DaCosta . 2021. “Challenges Facing the Nursing Profession in Saudi Arabia: An Integrative Review.” Nursing Reports 11, no. 2: 395–403. 10.3390/nursrep11020038. 34968216 PMC8608082

[jan16875-bib-0019] Alshahrani, S. H. 2022. “Reasons, Consequences, and Suggested Solutions for Nursing Workforce Shortage: A Review of the Literature.” International Journal of Health Sciences 6, no. S5: 1557–1568.

[jan16875-bib-0020] Alshareef, A. G. 2019. “Identifying Factors Influencing Saudi Arabian Nurse Turnover.” Doctor of Philosophy, Queensland University of Technology. https://eprints.qut.edu.au/130634/9/Abdullah%20Ghaleb%20S%20Alshareef%20Thesis.pdf.

[jan16875-bib-0021] Alshareef, A. G. , D. Wraith , K. Dingle , and J. Mays . 2020. “Identifying the Factors Influencing Saudi Arabian Nurses' Turnover.” Journal of Nursing Management 28, no. 5: 1030–1040. 10.1111/jonm.13028. 32277535

[jan16875-bib-0022] Alsufyani, A. M. , M. A. Alforihidi , K. E. Almalki , S. M. Aljuaid , A. A. Alamri , and M. S. Alghamdi . 2020. “Linking the Saudi Arabian 2030 Vision With Nursing Transformation in Saudi Arabia: Roadmap for Nursing Policies and Strategies.” International Journal of Africa Nursing Sciences 13: 100256. 10.1016/j.ijans.2020.100256. 33072514 PMC7553899

[jan16875-bib-0023] Alzahrani, M. S. J. 2022. “Impact of Work Environment on Nurse's Retention at Hospital: Scoping Review.” Evidence‐Based Nursing Research 4, no. 2: 15.

[jan16875-bib-0024] Booth, A. , J. Noyes , K. Flemming , et al. 2018. “Structured Methodology Review Identified Seven (RETREAT) Criteria for Selecting Qualitative Evidence Synthesis Approaches.” Journal of Clinical Epidemiology 99: 41–52. 10.1016/j.jclinepi.2018.03.003. 29548841

[jan16875-bib-0025] Burnard, P. , P. Gill , K. Stewart , E. Treasure , and B. Chadwick . 2008. “Analysing and Presenting Qualitative Data.” British Dental Journal 204, no. 8: 429–432. 10.1038/sj.bdj.2008.292. 18438371

[jan16875-bib-0026] Cardiff, S. , O. Gershuni , and A. Giesbergen‐Brekelmans . 2023. “How Local, First‐Line Nurse Leaders Can Positively Influence Nurse Intent to Stay and Retention: A Realist Review.” Journal of Clinical Nursing 32, no. 19–20: 6934–6950. 10.1111/jocn.16813. 37421611

[jan16875-bib-0027] Clemmons‐Brown, C. A. 2023. “Innovation and Evidence‐Based Decision‐Making: Addressing New Graduate Nurse Turnover.” Nursing Administration Quarterly 47, no. 1: E1–E11. 10.1097/NAQ.0000000000000567. 36469379

[jan16875-bib-0028] Cooke, A. , D. Smith , and A. Booth . 2012. “Beyond PICO: The SPIDER Tool for Qualitative Evidence Synthesis.” Qualitative Health Research 22, no. 10: 1435–1443. 10.1177/1049732312452938. 22829486

[jan16875-bib-0029] Cozart, A. 2024. “Effective Strategies to Reduce Registered Nurse Turnover in Hospitals.” Walden Dissertations and Doctoral Studies. 15726. https://scholarworks.waldenu.edu/dissertations/15726.

[jan16875-bib-0030] De Brún, A. , R. O'Donovan , and E. McAuliffe . 2019. “Interventions to Develop Collectivistic Leadership in Healthcare Settings: A Systematic Review.” BMC Health Services Research 19: 72. 10.1186/s12913-019-3883-x. 30683089 PMC6347820

[jan16875-bib-0031] Drennan, V. M. , and F. Ross . 2019. “Global Nurse Shortages‐The Facts, the Impact and Action for Change.” British Medical Bulletin 130, no. 1: 25–37. 10.1093/bmb/ldz014. 31086957

[jan16875-bib-0032] Falagas, M. , E. Pitsouni , G. Malietzis , and G. Pappas . 2008. “Comparison of PubMed, Scopus, Web of Science, and Google Scholar: Strengths and Weaknesses.” FASEB Journal 22, no. 2: 338–342. 10.1096/fj.07-9492lsf. 17884971

[jan16875-bib-0033] Falatah, R. 2021. “The Impact of the Coronavirus Disease (COVID‐19) Pandemic on Nurses' Turnover Intention: An Integrative Review.” Nursing Reports 11, no. 4: 787–810. 10.3390/nursrep11040075. 34968269 PMC8715458

[jan16875-bib-0034] Falatah, R. , and E. Conway . 2019. “Linking Relational Coordination to Nurses' Job Satisfaction, Affective Commitment and Turnover Intention in Saudi Arabia.” Journal of Nursing Management 27, no. 4: 715–721. 10.1111/jonm.12735. 30449053

[jan16875-bib-0035] Falatah, R. , and O. A. Salem . 2018. “Nurse Turnover in the Kingdom of Saudi Arabia: An Integrative Review.” Journal of Nursing Management 26, no. 6: 630–638. 10.1111/jonm.12603. 29624760

[jan16875-bib-0036] Flemming, K. , A. Booth , R. Garside , Ö. Tunçalp , and J. Noyes . 2019. “Qualitative Evidence Synthesis for Complex Interventions and Guideline Development: Clarification of the Purpose, Designs and Relevant Methods.” BMJ Global Health 4, no. Suppl 1: e000882. 10.1136/bmjgh-2018-000882. PMC6350756 30775015

[jan16875-bib-0037] Gehanno, J.‐F. , L. Rollin , and S. Darmoni . 2013. “Is the Coverage of Google Scholar Enough to Be Used Alone for Systematic Reviews.” BMC Medical Informatics and Decision Making 13: 7. 10.1186/1472-6947-13-7. 23302542 PMC3544576

[jan16875-bib-0038] Karimi‐Shahanjarini, A. , E. Shakibazadeh , A. Rashidian , et al. 2019. “Barriers and Facilitators to the Implementation of Doctor‐Nurse Substitution Strategies in Primary Care: A Qualitative Evidence Synthesis. The.” Cochrane Database of Systematic Reviews 2019, no. 4: CD010412. 10.1002/14651858.CD010412.pub2. PMC6462850 30982950

[jan16875-bib-0039] Kingdom of Saudi Arabia . 2017. “Saudi Vision 2030.” Vision 2030. http://vision2030.gov.sa/en.

[jan16875-bib-0040] Lewin, S. , M. Bohren , A. Rashidian , et al. 2018. “Applying GRADE‐CERQual to Qualitative Evidence Synthesis Findings—Paper 2: How to Make an Overall CERQual Assessment of Confidence and Create a Summary of Qualitative Findings Table.” Implementation Science: IS 13, no. Suppl 1: 10. 10.1186/s13012-017-0689-2. 29384082 PMC5791047

[jan16875-bib-0041] Long, H. , D. French , and J. Brooks . 2020. “Optimising the Value of the Critical Appraisal Skills Programme (CASP) Tool for Quality Appraisal in Qualitative Evidence Synthesis.” Research Methods in Medicine & Health Sciences 1, no. 1: 31–42. 10.1177/2632084320947559.

[jan16875-bib-0042] Malpass, A. 2009. ““Medication Career” or “Moral Career”? The Two Sides of Managing Antidepressants: A Meta‐Ethnography of Patients' Experience of Antidepressants.” Social Science & Medicine 68, no. 1: 154–168. 10.1016/j.socscimed.2008.09.068. 19013702

[jan16875-bib-0043] Noyes, J. , A. Booth , M. Cargo , et al. 2018. “Cochrane Qualitative and Implementation Methods Group Guidance Series—Paper 1: Introduction.” Journal of Clinical Epidemiology 97: 35–38. 10.1016/j.jclinepi.2017.09.025. 29242094

[jan16875-bib-0044] Perkins, A. 2021. “Nursing Shortage: Consequences and Solutions.” Nursing Made Incredibly Easy 19, no. 5: 49. 10.1097/01.NME.0000767268.61806.d9.

[jan16875-bib-0045] Saleh, U. , T. O'Connor , H. Alsubhi , R. Alkattan , S. Al‐Harbi , and D. Patton . 2018. “The Impact of Nurse Managers' Leadership Styles on Ward Staff.” British Journal of Nursing 27: 197–203. 10.12968/bjon.2018.27.4.197. 29457941

[jan16875-bib-0046] Shatnawi, R. 2020. “Perceived Job Stress and Job Satisfaction Among Intensive Care Nurses in the Kingdom of Saudi Arabia.” Doctor of Philosophy. Anglia Ruskin University.

[jan16875-bib-0047] Soqair, N. Y. A. 2021. “Factors Affecting Nurses' Turnover in Alhassa Governmental Hospitals.” Open Journal of Nursing 11, no. 11: 960–980. 10.4236/ojn.2021.1111078.

[jan16875-bib-0048] Suliman, M. , M. Aljezawi , S. Almansi , A. Musa , M. Alazam , and W. F. Ta'an . 2020. “Effect of Nurse Managers' Leadership Styles on Predicted Nurse Turnover.” Nursing Management (Harrow, London, England: 1994) 27, no. 5: 20–25. 10.7748/nm.2020.e1956. 32662259

[jan16875-bib-0049] Xu, G. , X. Zeng , and X. Wu . 2023. “Global Prevalence of Turnover Intention Among Intensive Care Nurses: A Meta‐Analysis.” Nursing in Critical Care 28, no. 2: 159–166. 10.1111/nicc.12679. 34261191

[jan16875-bib-0050] Yun, M. R. , and B. Yu . 2021. “Strategies for Reducing Hospital Nurse Turnover in South Korea: Nurses' Perceptions and Suggestions.” Journal of Nursing Management 29, no. 5: 1256–1262. 10.1111/jonm.13264. 33486834

