# Supplementary material for: Turnover and Turnover Intention Among Nurses Working in Saudi Arabia: A Qualitative Evidence Synthesis
Source: J Adv Nurs. 2025 Mar 14;81(12):8513–28. doi: 10.1111/jan.16875 (PMC12623702; doi:10.1111/jan.16875)
Supplement: Supplementary file 1 — Table S1. [file JAN-81-8513-s001.docx]

**Supplementary Table: Characteristics of included studies**

| **ID** | **First Author** | **Year** | **Participant Characteristics** |
| --- | --- | --- | --- |
| 1 | Alotaibi | Article: (2016) | Area/Unit of Work: Not mentioned. Years of Work: Not mentioned. Designation: Not mentioned. |
| 2 | Almansour | Thesis:  (2017) Article:  (2022) | Area/Unit of Work: Not mentioned. Years of experience: 19.2% (<5 years), 34.6% (5-10 years), 46.2% (>10 years).  Designation: Not mentioned. |
| 3 | Aljohani | Article:  (2018) | Area/Unit of Work: 22.86% Medical/surgical, 22.86% Cardiac centre, 11.43% Oncology centre, 14.29% Emergency room, 28.57% Intensive care unit.  Years of Work: 1-15 years.  Designation: Ward staff. |
| 4 | Saleh | Article:  (2018) | Area/Unit of Work: 80.6% hospitals, 19.4% primary healthcare. Years of Work: 50% (1-3 years), 31.4% (4-6 years), 18.5% (>6 years). Designation: Not mentioned. |
| 5 | Alshareef | Thesis:  (2019) Article:  (2020) | Area/Unit of Work: Not mentioned. Years of Work: Not mentioned. Designation: Not mentioned. |
| 6 | Shatnawi | Thesis:  (2020) | Area/Unit of Work: Intensive and critical care units in two teaching hospitals. Years of Work: 36.9% (1-5 years), 52.6% (6-10), 10.5% (>10 years). Designation: Not mentioned. |
| 7 | Al-Nusair | Article:  (2022) | Area/Unit of Work: 5 medical surgical units and spinal cord injury units, 5 emergency department, hemodialysis unit, and outpatient clinics, 5 intensive care unit, pediatric unit, and operation room, 5 brain injury, women's health, and stroke units. Years of Work: The average years of experience were 10.0 years (SD = 6.9 years). Designation: Staff nurses and charge nurses. |
